# What Do We Really Know about How CD4 T Cells Control *Mycobacterium tuberculosis*?

**DOI:** 10.1371/journal.ppat.1002196

**Published:** 2011-07-28

**Authors:** Egidio Torrado, Andrea M. Cooper

**Affiliations:** Trudeau Institute, Inc., Saranac Lake, New York, United States of America; University of Washington, United States of America

Two recent papers in *PLoS Pathogens* have investigated the activity of antigen-specific cells within the lung of mice infected with *Mycobacterium tuberculosis* (Mtb) [1], [2]. To the uninitiated this may seem to be redundant, as “we all know” that antigen-specific cells make interferon gamma (IFNγ) and tumor necrosis factor (TNF), which activate infected phagocytes to kill the bacteria. However, what we really know is that IFNγ and TNF are essential for controlling both bacterial growth and immunopathology and that the acquired immune response is critical to orchestrating immunity [3]. What we also know is that cessation of bacterial growth in the lungs correlates temporally with the accumulation of IFNγ-producing antigen-specific T cells in the infected lung [3]. But several questions have been circulating in the field for some time, including, What are effector T cells doing during tuberculosis to mediate protection, and How does the environment within the granuloma affect this activity? [3].

In the first of these stimulating *PLoS Pathogens* papers, Gallegos et al. provided compelling evidence that CD4 T cells can induce Mtb growth arrest, even when unable to secrete IFNγ, TNF, or both cytokines [1]. In the second paper, Bold et al. showed that CD4 T cell activation (as measured by production of IFNγ) is suboptimal in the lungs of infected animals, and they suggest that this contributes to the inability of the host to eliminate the infection; they also link this low frequency of T cell activation to the level of cognate antigen in the lung [2].

In the paper by Gallegos et al., they investigated the relevance of cytokine-producing CD4 T cells during experimental Mtb infection by the transfer of T cell receptor transgenic (TCR Tg) cells into host mice. They found that growth arrest over the first 21 days after aerosol challenge occurred even when these cells were unable to express the Th1-promoting transcription factor T-bet or to secrete IFNγ, TNF, or both cytokines [1]. Of equal importance was the fact that there was no need for host IFNγ, TNF, the inducible nitric oxide synthase (iNOS) gene, or superoxide-generating machinery to mediate this control [1]. This antigen-specific effect could be seen both with in vitro expanded and polarized T cells as well as, to a lesser degree, naive T cells [1]. In addition, the authors showed that this was not a property of all in vitro–generated cells, since Th2 differentiated cells could not induce Mtb growth arrest as efficiently as Th1 or even Th17 differentiated cells. While the mechanism was not identified, previously published data have shown that in vitro–generated memory CD4 T cells enhance protection in the flu model by induction of multiple innate cytokines and chemokines in the lung in an antigen-dependent, but IFNγ and TNF independent, manner [4]. Another possibility is that elevated precursor frequency may dampen the activation of antigen-specific regulatory T cells, and indeed, the authors report that the adoptively transferred cells delayed the priming of the endogenous response [1]. Recent data have shown that regulatory T cells are induced very early and can regulate effector function in the aerosol Mtb models [5]. Bold et al. considered the importance of competition in their study but were concerned that the endogenous response was limiting the transferred response as a result of competition or regulatory activity—they found, however, that depletion of half of the endogenous cells within the lung did not result in increased activation of the transferred effector cells [2].

The “take home” message of the Bold paper is that the frequency of cells that produce IFNγ is low, even at the peak of the response, and that it decreases during the chronic phase [2], supporting previous work that suggested this pattern [6]. In the Bold paper, just as in the Gallegos paper, the authors transferred pre-activated antigen-specific TCR Tg CD4 T cells and used the expression of IFNγ (assessed directly ex vivo) as a marker of antigen recognition. They showed that the frequency of IFNγ-producing cells correlated with the availability of cognate antigen and that delivery of the cognate peptide resulted in greatly increased frequency of cytokine expression [2]. They also saw a modest decrease in bacterial numbers when the antigen was either forcibly expressed by the Mtb or if the antigen was delivered exogenously to the infected mice. Initiation of CD4 T cell responses during tuberculosis occurs in the lung-draining lymph nodes rather than in the lung; however, the data by Bold et al. support the hypothesis that CD4 T cells need to see antigen once again within the infection site to express their effector function. Another recent paper, wherein intravital multiphoton imaging was used to compare the movement patterns and effector function of pre-activated and control TCR Tg CD4 T cells (from the p25 mouse specific for Ag85 [7] also used in [2]), has also shown that effector function is poorly expressed in the granuloma [8]. The authors made the surprising observations that both mycobacteria-specific and non-specific CD4 T cell migrated vigorously through the granuloma, with very few antigen-specific T cells showing migration arrest, a hallmark of potent antigen recognition and presentation. Despite the observed rapid migration, the antigen-specific cells reacted differently to the antigen in the environment as they upregulated CD69, whilst the control non-antigen-specific T cells did not. As was seen in the Bold paper, there was very little real-time expression of IFNγ within the granuloma in this model. These data were taken to reflect the failure of the available antigen to signal both migration arrest and cytokine production by the effector cell, and this was supported by the fact that delivery of exogenous peptide resulted in expression of these functions by the antigen-specific cells within the granuloma [8]. One exciting observation of this work was that the cells that did exhibit migration arrest could (but not always) produce a targeted release of cytokine to a closely adjacent infected cell; these data suggest that the accepted protective mechanism of T cell–derived IFNγ-mediation of infected phagocyte activation does occur in the granuloma.

**Figure 1:**
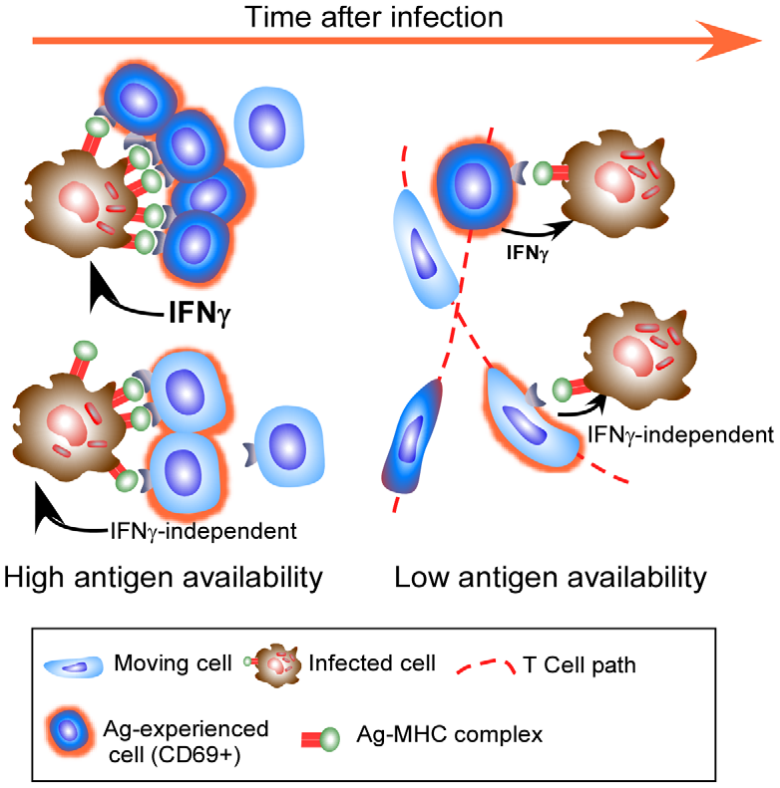
Effector T cells do not find the granuloma to be a stimulating environment. Effector T cells enter the granuloma and only a few exhibit significant migration arrest (dark blue cells) and targeted release of IFNγ, likely when they encounter a high level of cognate antigen on infected phagocytes. As their cognate antigen is reduced, even fewer cells undergo migration arrest, with many more cells continuing to move throughout the granuloma (light blue motile cells). Although these cells do not stop migrating, they do up regulate CD69 in an antigen-specific manner. Cells entering the granuloma may mediate their effector function without the release of IFNγ, and while this activity does require recognition of antigen, it may not need migration arrest.

However, putting these recent observations together, it is clear that the accepted mechanism may not be all there is to the control of Mtb. It would seem that expression of full effector activity by antigen-specific CD4 T cells within the granuloma is constrained by antigen availability and that there is the potential for antigen-specific T cells to mediate their effector function without the use of cytokine. As we cannot currently measure this effector function, other than by bacterial arrest, we cannot discount the possibility that the appropriate effector function is being expressed, but that it does not require significant migration arrest or cytokine production (Figure 1).

Other factors to take into account when thinking about the above papers is the artificial nature of transferred TCR Tg T cells, which may allow them to act differently to endogenous responses. It is also important to remember that specific protective immune mechanisms have different levels of importance depending on the potency of the bacterial challenge (discussed in [9]). Most importantly, the relative levels of specific mycobacterial antigens, particularly Ag85, which is the target of the TCR Tg cells used to assess effector function in the granuloma [2], [8], change over time as a function of bacterial physiology in the face of host immunity [10], [11]. These changes in bacterial activity will substantially impact the readout to any one antigen, and it is therefore important to investigate the activity of cells specific for other antigen as well as to measure activities other than IFNγ production. Despite the caveats, these papers remind us that although bacterial growth ceases within the resistant mouse model, we still do not know quite how this occurs. These recent excellent papers prompt us to continue to investigate the microanatomy of T cell function within the granuloma and to not be content with “what we know”.

Finally, the key question is, how can this information improve control of tuberculosis? It is certainly critical to define the protective effector functions of antigen-specific T cells as well as to determine the significance of suboptimal T cell activation. In this way we will be better able to design more effective vaccines and to determine whether the limited T cell activation in the granuloma is a host mechanism to cope with chronic infection or a mechanism Mtb evolved to prevent elimination by the host [12]. By continuing to pursue the above goals, we will be able to manipulate T cell responses in the infection site to enhance their effector function and to tip the balance of disease in favor of the host with minimal immunopathological consequences.
